# Effectiveness analysis of spatially targeted mollusciciding for *Oncomelania* snail control

**DOI:** 10.1017/S0950268820001466

**Published:** 2020-07-01

**Authors:** Y. Xie, W. Wu, Y. Zhang, Y. Zhang, Z. Peng, Y. Hu, M. Xu, X. Tan

**Affiliations:** 1 School of Health Sciences, Wuhan University, Wuhan, China; 2 School of Public Health and Management, Hubei University of Medicine, Shiyan, China; 3 Disease Prevention and Control Centre of Wuhan City Jiang'an District, Wuhan, China; 4 Wuhan Centre for Disease Prevention and Control, Wuhan, China; 5 School of Nursing, Wuchang University of Technology, Wuhan, China

**Keywords:** Control, infectious disease, parasitic disease epidemiology and control, public health, schistosomiasis (bilharziasis)

## Abstract

A new developed spatially targeted mollusciciding technology for snail control was utilised in a research site. This study aims to analyse whether this technology can achieve rational effectiveness compared with the routine method. Snail density was monitored every spring and autumn from 2010 to 2017 at the research site and routine mollusciciding for snail control was then performed. After snail density monitoring in spring 2018, spatially targeted mollusciciding technology was adopted. Log-linear regression and nonlinear regression models were used for snail density prediction in autumn 2018 and the predicted value was compared with the actual snail density in autumn 2018 to verify the effectiveness of the spatially targeted mollusciciding. Monitoring results showed that overall snail density in the research site decreased from 2010 to 2018. The monitored snail density in autumn 2018 was 0.014/0.1 m^2^. Predicted by the log-linear regression model, the snail density in autumn 2018 would be 0.028 (95% CI 0.11–0.072)/0.1 m^2^. Predicted by the nonlinear regression model, the snail density growth in autumn 2018 in contrast to spring 2018 would be 79.79% (95% CI 54.81%–104.77%) and the actual value was 55.56%. Therefore, the effectiveness of the first application of spatially targeted mollusciciding was acceptable. However, the validation of its sustainable effectiveness still needs a replicated study comparing areas where targeted and untargeted methods are applied simultaneously and both snail abundance and human infection are monitored.

## Introduction

Schistosomiasis is an acute and chronic parasitic disease caused by blood flukes from the genus *Schistosoma*. It is prevalent in tropical and subtropical areas and is related to poverty, especially in poor communities without access to safe drinking water and adequate sanitation [1]. Schistosomiasis transmission has been reported in 78 countries with over 250 million people being infected currently, and 800 million people in endemic areas are at risk of infection, making the burden of disease reach 70 million disability-adjusted life years [2].

According to the strategies recommended by the World Health Organization (WHO), the control of schistosomiasis should focus on the large-scale treatment of at-risk population groups, simultaneously, a more comprehensive approach, including safe water, adequate sanitation, hygiene education and snail control, is also essential to reducing transmission [3], i.e. schistosomiasis eradication must simultaneously involve chemotherapy treatment and transmission control. Snail control is critical to transmission control and historically, elimination of schistosomiasis has been more likely when snail control was used [4].

During the 20th century, molluscicide application was the most commonly used snail control strategy [5]. Though molluscicide application has been limited due to its environmental impacts, it is also effective in snail control [6]. At present, new snail control strategies, such as environmental diagnostics or transformation, gene drive technologies, traps, natural enemies and decoys, have emerged. However, ecological engineering requires a relatively large capital investment. Biological techniques are lacking in mechanism research and their large-scale application is still in the experimental stage to a certain extent. On this basis, using molluscicide, especially chemically synthesised drugs is still the main method of snail control [7].

In China, schistosomiasis has been reported in the middle and lower reaches of the Yangtze River and south of its basin. By the end of 2016, a total of 451 endemic counties (cities, districts) covering 257 million people were affected. Among these counties, a total of 29 692 endemic villages with 69.39 million people were at risk of developing schistosomiasis. In addition, the snail distribution area in China is large and the infection source of schistosomiasis still exists in some endemic areas [8].

The city of Wuhan, Hubei Province, China, is located in the middle reaches of the Yangtze River. In recent years, the monitoring results of the local Centre for Diseases Control and Prevention (CDC) showed that scattered snail distribution has always existed in some riverfront. Thus, chemical treatment was adopted for snail control after the annual snail monitoring work. The chemically synthesised drug niclosamide which was recommended by the WHO for snail control [9], was applied as molluscicide. According to the Chinese CDC (CCDC) [10], four molluscicide application methods can be used: (1) drug solution immersing, (2) drug sprinkling and sod scalping along the edges, (3) drug solution spraying and (4) drug powder spraying. Selecting which method depends on the environment of the snail region. In large riverfront areas, the water source is abundant. Thus, the drug solution spraying method was mainly adopted by the local CDC.

In routine snail control work, molluscicide application area was zoned on the basis of snail distribution unit according to snail monitoring and the drug solution was sprayed throughout the area. However, this molluscicide application method must be improved because (1) the snail distribution has become scattered and the snail density has considerably declined and (2) the application of precise positioning technology during snail survey can determine the location of snails. Therefore, molluscicide application covering a large area is inefficient and requires additional manpower, working hours and molluscicide consumption, which will increase the environmental load.

After snail monitoring work in Jiang'an District of Wuhan in spring 2018, a part of Zhujia River beach with scattered snail distribution was selected as the research site and a newly developed molluscicide application technology, namely, spatially targeted mollusciciding was utilised. This study analysed the effectiveness of this new technology.

## Methods

### Study design

**Fig. 1.**
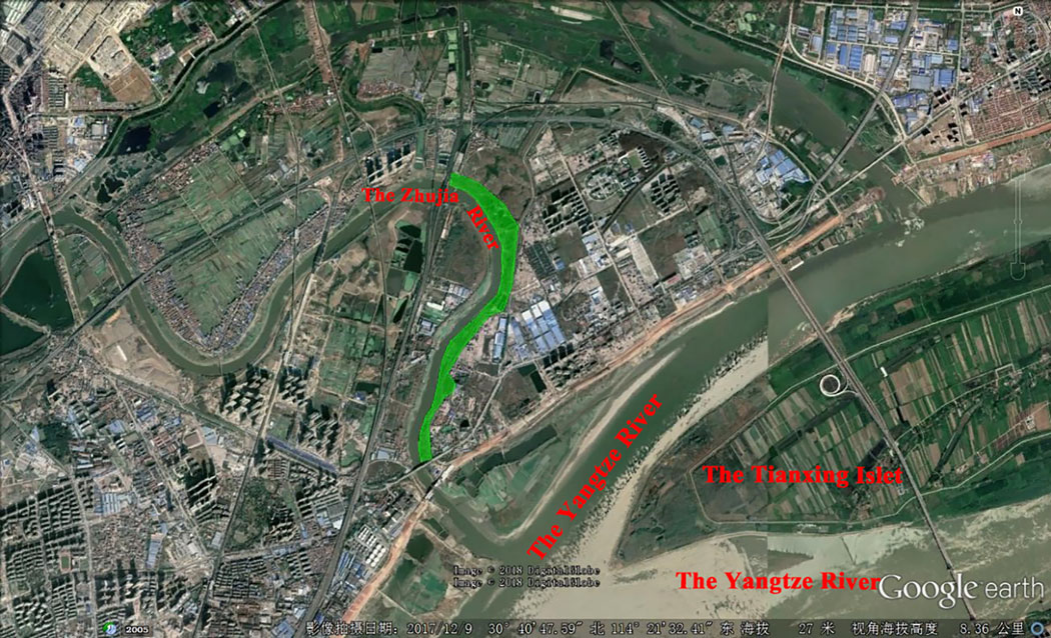
Research site of spatially targeted mollusciciding. Green shaded area, which was selected as the research site, is a part of the northern riverfront of the Zhujia River. Two bridges over the river and the embankment are the boundaries of this area.

From 2010 to 2018, snail monitoring work was conducted twice every year, namely, April (spring) and September (autumn), in snail-infested areas in Jiang'an District of Wuhan and snail control work was then performed on the basis of snail distribution. After snail monitoring in spring 2018, a part of the riverfront of Zhujia River (Fig. 1) with scattered snail distribution was selected as the research site and the newly developed spatially targeted mollusciciding was implemented. Scattered snail distribution in this study was defined as: among all the distances between every two nearest snail points, more than 80% were greater than 100 m. The field study was conducted in accordance with the local legislation and obtained the approval of Wuhan Municipal Health Committee.

Considering different environmental conditions, such as hydrology and main vegetation of the riverfront, which could affect the change in snail density between adjacent areas and the research site [11], we used the research site for comparison to avoid biases that are caused by these factors as much as possible. We attempted to establish a hypothetical snail density for autumn 2018 through prediction， and the predicted value was used as an estimate of what would have happened if the previous regime of routine mollusciciding were continued. On the basis of snail densities of 2010–2017, two methods were used for predicting snail density in autumn 2018. The log-linear regression model was based on the autumn snail densities of 2010–2017 and the nonlinear regression model was based on the autumn snail density growth relative to spring from 2010 to 2017.

In autumn 2018, snail density was continuously monitored and the predicted value was compared with the actual snail density to verify the effectiveness of the new snail control technology. Molluscicide application area, molluscicide consumption, working hours and cost of routine mollusciciding were also calculated and compared with those of spatially targeted mollusciciding based on the actual snail distribution.

### Snail monitoring

Snail monitoring was conducted using a handheld Global Position System (GPS). Systematic sampling method was used to determine the survey unit, which was a square of 0.1 m^2^ in area with a side length of 33 cm frame. When conducting the systematic sampling, a total of six survey lines were set on the riverfront, parallel to the river direction and each survey line was 20 m apart depending on the width of the riverfront. For each survey line, two investigators were present and a survey unit was set every 10 m. During the field investigation, each survey unit was marked in the GPS and the number of snails per unit was also simultaneously recorded. The geographic information system and Google Earth software were then used to pinpoint the snail locations on the digital map, in which their geographical features and the present overall snail distribution were described. The living snail density was defined as:
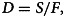
where *S* and *F* are the number of living snails and survey units, respectively [12].

### Spatially targeted mollusciciding

Processed by Google Earth and Route Converter Windows software, the data with only snail point information was preserved and transferred back to the handheld GPS. On the map of the handheld GPS, the precise location of each snail point could be clearly shown. At the snail control worksite, the staff member first reached the snail point under the guidance of handheld GPS and then sprayed the drug solution within a 50 m radius with the snail point as the centre. A 50% niclosamide ethanolamine salt wettable powder was used. The dosage at each single snail point was 15.7 kg (2 g/m^2^) and the water consumption was 7.8 m^3^ (1 L/m^2^). The powder was evenly mixed before spraying. If the distance of snail points was within 100 m, the drug solution spraying should cover the entire snail area that was delimited in accordance with the guidelines of CCDC [10].

### Log-linear regression model

Due to snail control work carried out by the local CDC every year, snail density of the research site showed a downward trend overall from 2010 to 2017. Theoretically, the population density of snail could affect its reproduction rate and snails with low population density have a high reproductive rate [13]. Thus, the decline rate of snail density would decrease with the lower level of snail density and the snail density would reach a plateau at a low value near zero. Accordingly, we adopted an exponential decay process to describe the change of snail density with time and their relationship was as follows:
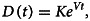
where *t* was the ordinal number of years, *K* and *V* were coefficients of the equation. The log-linear regression model was obtained by the logarithm change of this equation:
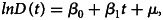
where *β*_0_ was the intercept, *β*_1_ was the slope and *μ* was the error.

### Nonlinear regression model

Snail monitoring result showed although the development of snail density varied in different seasons, the annual rate of snail density growth in the autumn relative to the spring was trending downward. According to the scatter plot of annual snail density growth, the power function was adopted as the basis for nonlinear regression modelling. The year was treated as a continuous variable and the nonlinear regression model was constructed as follows:
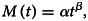
where *M* is the autumn snail density growth and *t* is the ordinal of a particular year. Multiple coefficients of determination (*R*^2^) were adopted to verify the accuracy of this regression model. The growth in autumn 2018 relative to spring was predicted by the model and then compared with the actual one.

### Statistical analysis

Log-linear regression and nonlinear regression analyses were conducted using the R software (version 3.5.1).

## Results

### Snail densities of research site from 2010 to 2018

**Fig. 2.**
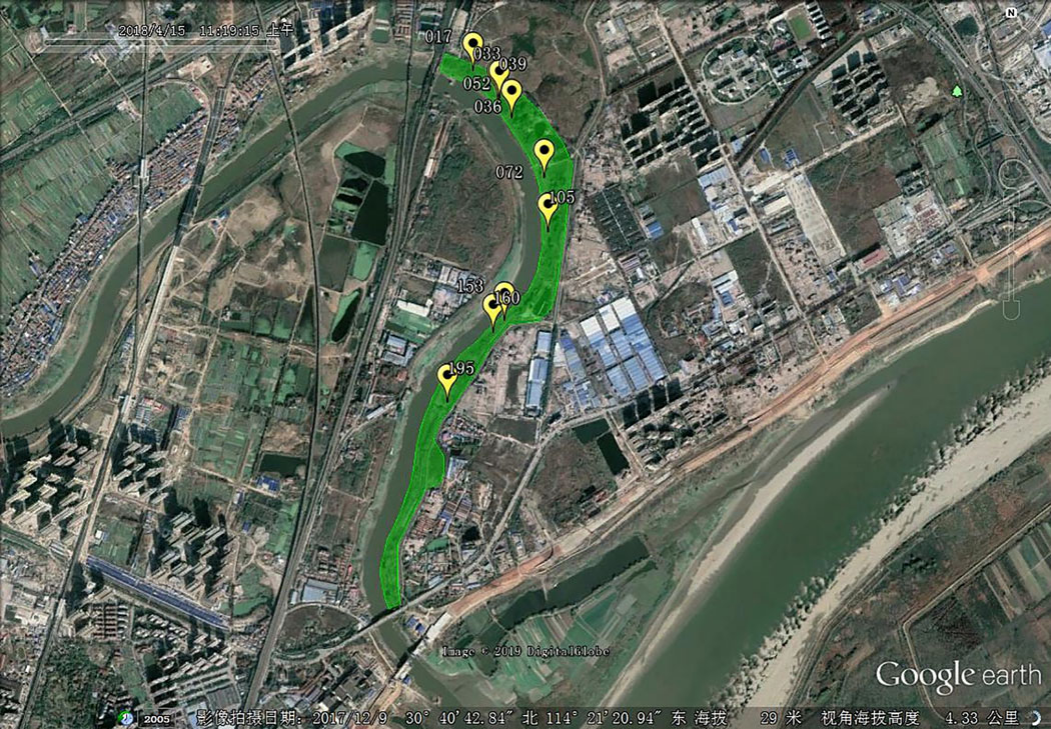
Snail points in research site in spring 2018. Green shaded area is the research site and the yellow marks are the survey units with living snail(s).

The spring and autumn snail densities from 2010 to 2018 in the research site in Wuhan City are shown in Table 1. Overall, the snail density decreased from 2010 to 2018. Both spring and autumn snail densities showed declining trends. The autumn snail density growth relative to spring presented varying values that were decreasing overall. Spatially targeted mollusciciding was applied on the basis of snail distribution in spring 2018, as displayed in Figure 2.

**Table 1.** Snail densities from 2010 to 2018 in research site of the Zhujia River

Year	Snail density (/0.1 m^2^)		Growth (%)
	Spring	Autumn	
2010	0.064	0.486	659.38
2011	0.090	0.335	272.22
2012	0.059	0.201	240.68
2013	0.086	0.188	118.60
2014	0.081	0.211	160.49
2015	0.074	0.197	166.22
2016	0.014	0.034	142.86
2017	0.015	0.028	86.67
2018	0.009	0.014	55.56

### Log-linear regression model

The established log-linear regression model was:
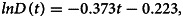


The estimate of *β*_1_ was significant (*P* < 0.001) and the standard error was 0.078. The analysis of variance showed *F* = 23.12 (*P* < 0.01). The multiple coefficient of determination (*R*^2^) of the equation was 0.794 and the adjusted *R*^2^ was 0.760.

According to the result predicted by the model, under the use of the routine mollusciciding method, the snail density in autumn of 2018 should be 0.028 (95% CI 0.011–0.072)/0.1 m^2^. In fact, the actual value was 0.014/0.1 m^2^, which was within the confidence intervals of the predicted value. Thus, the actual snail density was considered acceptable.

### Nonlinear regression model

**Fig. 3.**
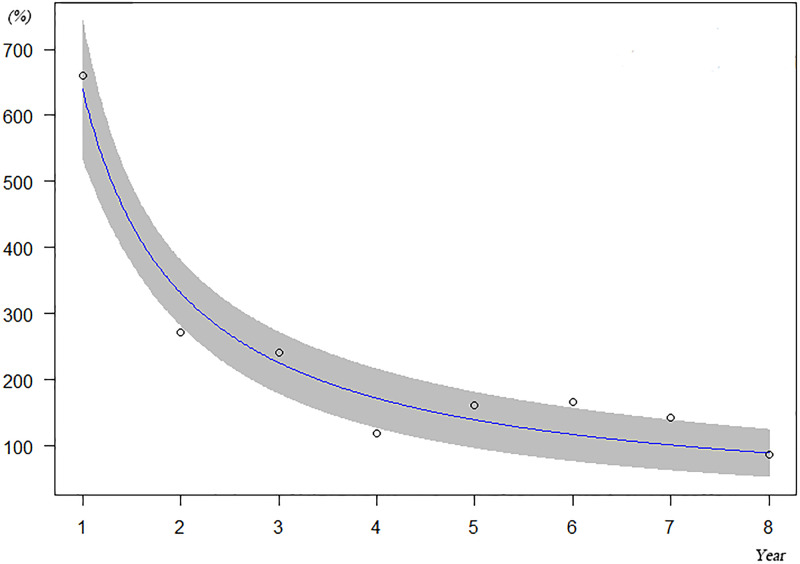
Fitting curve and its 95% CI of annual autumn snail density growth relative to spring from 2010 to 2017. The black dots are the actual value of annual autumn snail density growth relative to spring from 2010 to 2017. The blue curve is the fitting curve of the dots, the equation of which is *M* (*t*) = 639.199*t*^−0.947^. The grey shaded area is the 95% CI of the fitting curve.

The established nonlinear regression model was:
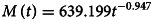


Thereinto, *a* (the estimate of parameter *α*) and *b* (the estimate of parameter *β*) were significant (*P* < 0.001) and the standard errors were 42.257 and 0.092, respectively. The multiple coefficients of determination (*R*^2^) of the equation were 0.951 and the adjusted *R*^2^ was 0.943. The fitting curve is presented in Figure 3.

On the basis of the model, the predicted snail density growth in autumn 2018 should be 79.79% (95% CI 54.81%–104.77%) and the actual value was 55.56%. This finding illustrated that compared with the routine method, the spatially targeted mollusciciding did not affect the decreasing trend of snail density in autumn 2018. The snail density was considered acceptable.

### Comparison of routine and spatially targeted mollusciciding

Table 2 displays the differences in the molluscicide application area, molluscicide consumption, working hours and cost between routine and spatially targeted mollusciciding in the research site. The items of routine mollusciciding were calculated on the basis of the actual snail distribution. The results showed that spatially targeted mollusciciding could reduce molluscicide application area compared with routine mollusciciding, thereby reducing molluscicide consumption, working hours and cost.

**Table 2.** Differences between routine and spatially targeted mollusciciding in the research site^a^

Items		Routine mollusciciding^b^	Spatially targeted mollusciciding	Reduction (%)
Molluscicide application area/m^2^		117 690	61 480	47.76
Molluscicide consumption/kg		235.38	122.96	47.76
Working hours/h		18	6	66.67
Cost/RMB	Manpower	5130	2040	60.23
	Molluscicide	10 592	5533	47.76
	Transportation^c^	2340	980	66.67
	Total	18 062	8553	52.65

a Only involved molluscicide application process, snail monitoring cost was not contained.

b The items of routine mollusciciding were calculated based on the actual snail distribution.

c Including vehicle transportation fee and vessel fuel fee.

## Discussion

In this study, spatially targeted mollusciciding for snail control was developed and applied in one research site. According to the results of snail density monitoring, the effectiveness of this technology, which was verified by log-linear regression and nonlinear regression models, was acceptable and the snail density was not runaway in a short period of time.

Schistosomiasis remains one of the major communicable diseases of public health and socioeconomic importance in the developing world [14]. Enhanced snail control will assist in achieving the goals set by the WHO of controlling morbidity in 2020 and eliminating schistosomiasis as a public health problem in certain regions by 2025 [15]. Chemical treatment is currently the main method of snail control due to its rapid effects [7]. In theory, the low molluscicide concentration is non-toxic to vertebrates, including fish and humans, and has no load on the environment for degradation; however, inevitable uneven dispersal during application might cause fish kills, health concerns and environmental contamination. Given the increased costs of chemicals and the possible development of snail resistance to molluscicide [16], mollusciciding technology must be improved.

Spatially targeted mollusciciding is a new method of molluscicide application that fully utilises the precise positional information of snail points obtained during snail monitoring; i.e., the spatial positioning of snails is first determined during the snail monitoring process. This method is essentially a targeted reduction in the area of molluscicide application. It could not theoretically improve the snail-killing effect of the routine method. The results showed that after the first use of spatially targeted mollusciciding, the snail density was within an acceptable range, which was predicted by both log-linear regression and nonlinear regression models. Thus, under constant dosage per unit area, spatially targeted mollusciciding could reduce the molluscicide application area, correspondingly saving molluscicide consumption, working hours, manpower and material cost. Furthermore, the less total dosage could lead to reduced environmental contamination. This advantage is only evident in environments with scattered snail distribution. Therefore, this method might be suitable only for areas where snail control has achieved certain effects.

In conclusion, the newly developed spatially targeted mollusciciding technology exhibited acceptable effectiveness during its first application. Given the reduction of molluscicide application area, this technology is environmentally and economically friendly, which might be appropriate for environments with scattered snail distribution.

Some limitations must be carefully considered in understanding the results. First, this study was a before-after comparison. We failed to establish parallel control groups for two main reasons: (1) an untreated control group could not be set because of the schistosomiasis control policy and ethical reasons (i.e. transmission control should be conducted in all areas with living snails). Therefore, the natural snail density was unascertainable. (2) We could not find an appropriate parallel control given that adjacent areas with snail distribution had different hydrology, vegetation and baseline snail density from those of the research site and these factors could affect the changes in snail density. Future study should continue to use spatially targeted mollusciciding in the research site and establish appropriate controls that perform routine mollusciciding. The control sites might be the riverfront of upstream with a reasonable distance from the research site. Second, given that this was the first application of the spatially targeted mollusciciding, it could not be conducted in many places simultaneously because of ethical issues. Thus, the sample size was small, which resulted in relatively wide confidence intervals. The preliminary findings of this study showed that additional areas with living snails should be included in the future study and continuous monitoring should be performed in succeeding years to observe whether the snails would bounce back over long time periods. The monitoring results should also be analysed in reference to the analysis of non-inferiority trials [17]. Third, the nonlinear regression model was established based on the scatter plot of the data， and although it fit the data well, a more plausible model may also exist. Future study should figure out the solid evidence of the change of annual snail density. Fourth, the optimum radius of drug application was not determined, which was set to 50 m only in this study. The distance setting was deemed conservative because it was the first attempt of spatially targeted mollusciciding. Finally, soil drug residues should be evaluated in future study to analyse the environmental effect of spatially targeted mollusciciding.

## Data Availability

The data that support the findings of this study are available from the corresponding author, X.T., upon reasonable request.
